# Development of a new alternative method to inhalation exposure: intratracheal instillation studies using molecular dispersion

**DOI:** 10.1265/ehpm.25-00142

**Published:** 2025-09-11

**Authors:** Toshiki Morimoto, Chinatsu Nishida, Hiroto Izumi, Taisuke Tomonaga, Kazuma Sato, Yasuyuki Higashi, Ke-Yong Wang, Takuma Kojima, Kazuo Sakurai, Akihiro Moriyama, Jun-ichi Takeshita, Kei Yamasaki, Hidenori Higashi, Kazuhiro Yatera, Yasuo Morimoto

**Affiliations:** 1 Department of Respiratory Medicine, University of Occupational and Environmental Health, Japan, 1-1 Iseigaoka, Yahata-nishi-ku, Kitakyushu, Fukuoka 807-8555, Japan; 2 Department of Environmental Health Engineering, Institute of Industrial Ecological Sciences, University of Occupational and Environmental Health, Japan, 1-1 Iseigaoka, Yahata-nishi-ku, Kitakyushu, Fukuoka 807-8555, Japan; 3 Department of Occupational Pneumology, Institute of Industrial Ecological Sciences, University of Occupational and Environmental Health, Japan, 1-1 Iseigaoka, Yahata-nishi-ku, Kitakyushu, Fukuoka 807-8555, Japan; 4 Shared-Use Research Center, School of Medicine, University of Occupational and Environmental Health, Japan, 1-1 Iseigaoka, Yahata-nishi-ku, Kitakyushu, Fukuoka 807-8555, Japan; 5 Department of Chemistry and Biochemistry, The University of Kitakyushu, 1-1 Hibikino, Wakamatsu-ku, Kitakyushu, Fukuoka 808-0135, Japan; 6 Research Institute of Science for Safety and Sustainability, National Institute of Advanced Industrial Science and Technology (AIST), 16-1 Onogawa, Tsukuba, Ibaraki 305-8569, Japan

**Keywords:** Polyacrylic acid (PAA), Organic compounds, Dispersion

## Abstract

**Background:**

Organic chemicals have been known to cause allergic diseases such as bronchial asthma and hypersensitivity pneumonitis; however, the possibility that they do not cause irreversible pulmonary fibrosis has not been considered. Polyacrylic acid (PAA), an organic chemical, has caused irreversible progressive pulmonary fibrosis in exposed workers, indicating its potential to induce pulmonary inflammation and fibrosis. Although intratracheal instillation studies are commonly used for evaluating lung pathology, traditional methods face challenges with chemical substances, particularly nanoparticles, which tend to aggregate in suspension and prevent uniform pulmonary distribution. Such aggregation alters the qualitative and quantitative responses to lung injury, limiting accurate assessment of lung pathology. To overcome this limitation, we developed a ‘molecular dispersion method’ that uses pH modification to negative charges to PAA particles, maintaining their dispersion. Using this method, we investigated the effects of PAA on pulmonary inflammation and fibrosis in a rat model.

**Methods:**

F344 rats were intratracheally instilled with PAA using molecular dispersion (0.1 mg/rat, 1.0 mg/rat), PAA without molecular dispersion (1.0 mg/rat), and normal saline (control group). Rats were sacrificed at 3 days, 1 week, 1 month, 3 months, and 6 months after exposure to examine inflammatory and fibrotic responses.

**Results:**

PAA caused persistent increases in neutrophil influx in the bronchoalveolar lavage fluid (BALF) from 3 days to 1 month following instillation. In histopathological findings, the group with molecular dispersion had almost no inflammatory masses in the lung tissue compared to the group without molecular dispersion, and exhibited relatively uniform dispersion.

**Conclusion:**

Intratracheal instillation of dispersed PAA induced neutrophil inflammation and fibrosis in the rat lung, suggesting that PAA might have pulmonary inflammogenicity and fibrogenicity. Intrapulmonary dispersion of PAA particles following intratracheal instillation studies using the molecular dispersion method was similar to that following inhalation studies.

**Supplementary information:**

The online version contains supplementary material available at https://doi.org/10.1265/ehpm.25-00142.

## 1. Introduction

Chemicals are classified into inorganic and organic substances. Among inorganic substances, asbestos and crystalline silica are known to cause lung diseases, including irreversible pulmonary fibrosis. On the other hand, organic substances cause allergic diseases such as bronchial asthma and hypersensitivity pneumonitis, but are not considered to cause pulmonary fibrosis directly. In recent years, however, it has been reported that organic chemicals induce progressive pulmonary fibrosis [1]. In 2017, it was reported that a high rate of progressive lung damage was observed among workers at a company manufacturing polyacrylic acid (PAA), an organic chemical [2]. PAA polymerizes through the repeated bonding of monomers, forming a polymer structure. The introduction of a crosslinking agent creates covalent bonds between multiple linear molecules, resulting in a more complex three-dimensional network structure.

PAA, an organic compound with water absorbency and thickening properties in aqueous solutions, is widely used in everyday products, ranging from diapers, shampoos, and cosmetics to food additives. Its extensive use in society is supported by its high safety profile [3, 4]. The progression of fibrosis is more rapid than that of lung disorders caused by asbestos or crystalline silica, which are known as representative inorganic chemicals with fibrogenicity. While asbestos-induced pneumoconiosis typically progresses slowly over 20 years or more after exposure [5], PAA-induced lung disorders develop within 2 to 3 years and progress substantially faster than pneumoconiosis caused by inorganic substances such as asbestos and crystalline silica [2, 6]. Due to this rapid progression, urgent investigation of its pathogenic mechanisms is required.

Intratracheal instillation is the most commonly used method for evaluating lung pathology. However, chemical substances, particularly nanoparticles, administered intratracheally aggregate in a suspension, preventing uniform distribution in the lungs and limiting the accurate assessment of lung pathology. Peng L reported that the aggregation of ceria nanoparticles changes the qualitative response of pulmonary inflammation [7]. Aggregation alters the quantitative inflammatory and fibrotic responses. Since nanoparticle aggregation may modify lung injury patterns, and humans typically inhale particles in a relatively dispersed state, it is essential to establish techniques for proper chemical substance dispersion. For that reason, we conducted intratracheal instillation using a method that ensures nanoparticle dispersion in the lungs.

This method involves changing the pH of the liquid to give the molecules of the chemicals in the suspension a negative charge, which repels the particles and inhibits their aggregation. We named this method the “molecular dispersion method.” In order to investigate lung disorder caused by PAA with the use of charged and dispersed PAA particles, we performed intratracheal instillation of PAA in a rat model and analyzed the pulmonary inflammation and fibrosis in the lung.

## 2. Materials and methods

### 2.1 Sample polymer

**Fig. 1:**
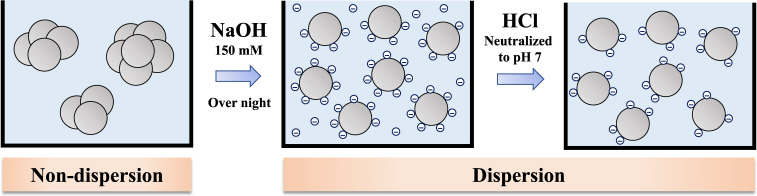
The novel molecular dispersion method used in this study. Substances are dissolved in NaOH solution (150 mM) and stirred overnight at room temperature. Then the pH is adjusted to about 7 by adding HCl solution gradually.

PAA samples were purchased from Sigma-Aldrich, St. Louis, Missouri, USA. PAA is a white, dispersible powder with a specific gravity of 1.2. For the molecular dispersion method, the operation was performed while measuring the pH of the solution with a pH meter (C-62, AS ONE, Tokyo, Japan). We dissolved PAA in a NaOH solution (150 mM) and stirred it overnight at room temperature, after which we adjusted the pH to about 7 by adding HCl solution little by little (Fig. 1).

### 2.2 Animals

Eight-week-old male Fischer 344 rats were purchased from Charles River Laboratories International, Inc., (Kanagawa, Japan). The animals were kept for acclimatization for 4 weeks at the Laboratory Animal Research Center of the University of Occupational and Environmental Health. They were kept under the same conditions as we previously described [6]. Briefly, they were kept with a light/dark 12 h/12 h cycle, 20–25 °C, 40–70% humidity with 15 times/hour, and had free access to commercial chow and water. All of the animal handling and procedures were carried out following the U.K. Animals (Scientific Procedures) Act, 1986, and the associated guidelines, EU Directive 2010/63/EU for animal experiments, and were approved by the Animal Care and Use Committee, University of Occupational and Environmental Health, Japan (animal studies ethics clearance proposal number: AE18-021).

### 2.3 Intratracheal instillation

Regarding the doses of PAA used in the present study, we calculated the amount of inhaled material accumulated in the lungs using the following formula: deposition rate × exposure concentration × exposure time (duration) × respiratory volume (which is the amount of PAA) = deposition fraction × exposure concentration of particle × exposure minutes in one day × days of exposure × tidal volume × breathing frequency [8]. Assuming a deposition fraction of 0.1, a rat tidal volume of 2.1 mL/time, and a respiratory rate of 102 times/min, the amounts of lung accumulation at exposure concentrations of 0.2 mg/m^3^ and 2.0 mg/m^3^ for 13 weeks of inhalation exposure were calculated as 0.1 and 1.0 mg, respectively [9]. This indicates that the dosage between inhalation exposure and single intratracheal instillation can be considered comparable. Rats (12 weeks old) received 0.1 mg (0.4 mg/kg BW) or 1.0 mg (4.0 mg/kg BW) of PAA suspended in 0.4 ml in single intratracheal instillations. The control group received normal saline, and a control group was established for each intratracheal installation. For the 1.0 mg non-dispersion group, PAA was dissolved in normal saline, while for the 1.0 mg dispersion group, PAA was dissolved using the molecular dispersion method. The control and exposure groups were randomly selected.

### 2.4 Animals following intratracheal instillation

Five rats were assigned to each exposure group (0.1 mg dispersion group [0.1 mg(d+)], 1.0 mg dispersion group [1.0 mg(d+)], and 1.0 mg non-dispersion group [1.0 mg(d−)]) and control group at 3 days, 1 week, 1 month, 3 months, and 6 months after intratracheal instillation. Animals were sacrificed at each time point under anesthesia with isoflurane (Pfizer Japan, Tokyo, Japan) inhalation. Body and lung weight were measured at autopsy. Blood was removed from the abdominal aorta, and the lungs were perfused with normal saline. The right lung was repeatedly infused with normal saline under a pressure of 20 cmH_2_O, following fluid recovery two times, while the left main bronchus was clamped. Between 5 and 13 mL of the recovered fluid (bronchoalveolar lavage fluid, BALF) was collected by free fall into tubes, and then the right and left lungs were separated. The third lobe of the right lungs was then stored at −80 °C, and the left lungs were inflated and fixed using 10% formaldehyde under a pressure of 25 cmH_2_O.

### 2.5 Cytospin analysis of inflammatory cells, and measurement of total protein lactate dehydrogenase (LDH) levels in bronchoalveolar lavage fluid (BALF)

The BALF was centrifuged at 400 g for 15 min at 4 °C, and the supernatant was transferred to a new tube to measure total protein and LDH levels. The pellets were washed by suspension in polymorphonuclear leukocyte (PMN) buffer (137.9 mM NaCl, 2.7 mM KCl, 8.2 mM Na_2_HPO_4_, 1.5 mM KH_2_PO_4_, 5.6 mM C_6_H_12_O_6_) and centrifuged at 400g for 15 minutes at 4 °C. After removal of the supernatant, the pellet was resuspended in 1 mL of PMN buffer. Total cell counts in the BALF were determined by ADAM-MC (AR BROWN CO., LTD., Tokyo, Japan). Cells were spread on glass slides using Cytospin (Cyto-Tek^®^ Centrifuge, Sakura Finetek Japan K.K., Tokyo, Japan) and stained with Diff-Quik (Sysmex CO., Kobe, Hyogo, Japan). The numbers of neutrophils and alveolar macrophages were examined by microscopic observation (BX50, OLYMPUS, Tokyo, Japan).

The concentration of protein in the BALF was measured by the Pierce™ 660 nm Protein Assay (Thermo Fisher Scientific Inc., Rockford, IL, USA) and estimated using a standard curve obtained from albumin standards (Thermo Fisher Scientific Inc., Rockford, IL, USA). The released LDH activity in the BALF supernatant was measured according to the instructions for use of the Cytotoxicity Detection KitPLUS (LDH) (Roche Diagnostics GmbH, Mannheim, Northrheim-Westfalen, Germany) and estimated by a standard curve obtained from known concentrations of recombinant LDH from rabbit muscle (Roche Diagnostics GmbH, Mannheim, North Rhine-Westphalia, Germany).

### 2.6 3D micro-CT imaging

A 3D micro-CT scan was performed hours to days before dissection at each observation point on three of the five animals in each group. The X-ray 3D micro-CT system (CosmoScan GX, Rigaku, Tokyo, Japan) was operated under the following conditions: scan time 4.0 minutes, average whole-body radiation dose 161.9 mGy/scan, tube voltage of 90 kV, tube current of 88 µA, and chest CT with a field of view (FOV) measuring 60 × 40 mm (voxel matrix: 512 × 512 × 512, voxel size: 120 × 120 × 120 µm). The rats were positioned in the prone posture during the scanning process, and sevoflurane (Pfizer, Tokyo, Japan) and oxygen were administered through a nose cone. The acquired images were retrospectively gated at both respiratory phases (inspiration and expiration).

### 2.7 Histopathology

The lung tissue fixed with formaldehyde was embedded in paraffin, sectioned to a thickness of 4 µm, and subjected to hematoxylin and eosin (HE) staining and Masson trichrome (MT) staining. Lung inflammation and fibrosis were examined using the inflammatory cell infiltration score [10] and the Ashcroft score [11], respectively, following previous reports. Briefly, the inflammatory cell infiltration score was obtained by scoring the degree of inflammatory cell infiltration in lung tissue as: none (0), minimal (0.5), mild (1), moderate (2), or severe (3). Scores were calculated using the following formula: [Σ (grade × number of animals with grade)]/total numbers of animals. The Ashcroft score was graded on a scale of 0 (normal lung) to 8 (most severe fibrosis) for lung fibrosis, and the grades were summed and then divided by the number of visual fields. Slides were evaluated for histologic changes by a certified pathologist.

### 2.8 Statistical analysis

Statistical analysis was carried out using IBM^®^ SPSS^®^ software (IBM Corporation, Chicago, IL, USA). *p* values <0.05 were considered statistically significant. Tukey’s honestly significant difference test was used to compare the mean values between the four groups: control, 0.1 mg dispersion, 1.0 mg dispersion, and 1.0 mg non-dispersion.

## 3. Results

### 3.1 Effect of pH change on dispersibility

**Fig. 2:**
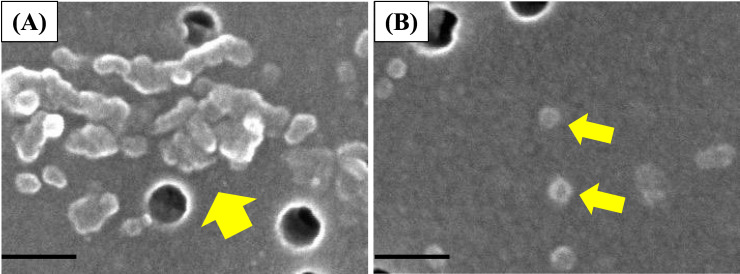
Scanning electron microscope (SEM) images of the polymers used in this study. **A** PAA suspended in distilled water. **B** PAA with using charged and dispersed PAA particles. Yellow arrows show PAA, and black bar is 100 nm.

**Fig. 3:**
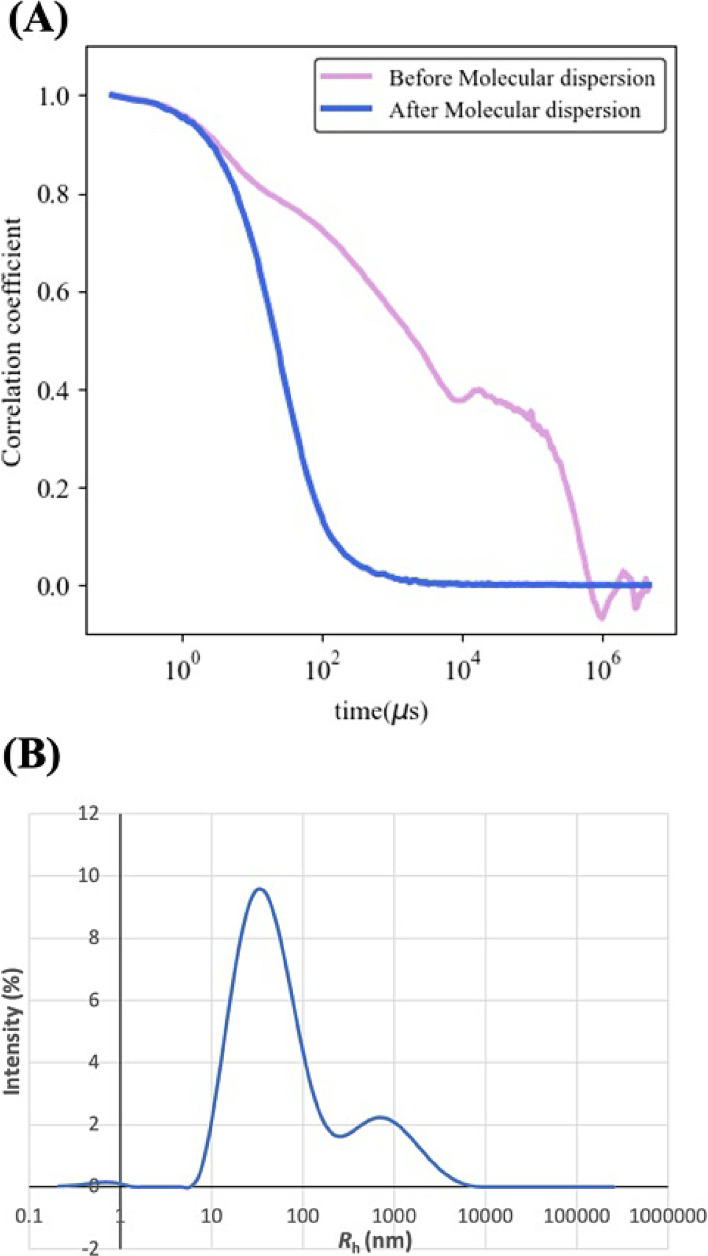
DLS (Dynamic Light Scattering) measures particle movement to determine size distribution through visualization technique. (A) The pink curve represents the state before molecular dispersion, while the blue curve represents the state after molecular dispersion. (B) The distribution shows particle intensity percentage versus hydrodynamic radius (Rh) in nanometers.

The fundamental characteristics of PAA are summarized in Table 1. The weight average molecular weight (Mw) is 7.68 × 10^5^, the number average molecular weight (Mn) is 4.14 × 10^5^, the polydispersity index (PDI) is 1.85, and the radius of gyration (Rg) is 68.7 nm, as measured by gel permeation chromatography (GPC) (Prominence 501 system, SHOKO SCIENCE, Kanagawa, Japan). Scanning electron microscope (SEM) images of the polymers used in this study, acquired with a HITACHI S-4500 (Hitachi, Tokyo, Japan) instrument, are shown in Fig. 2. Figure 2A and 2B show the PAA dispersed by being suspended in distilled water and by using molecular dispersion, respectively. In the 2.5 mg/mL suspension, particles exhibited a hydrodynamic radius of 0.11 µm with a mean error of 15.4 nm. The Zeta potentials of PAA suspended and dispersed in distilled water and of PAA dispersed using the molecular dispersion method were −2.08 mV and −22.9 mV, respectively. The relationship between particle movement and time for PAA before and after dispersion was analyzed by dynamic light scattering (DLS) (DelsaMax PRO, BECKMAN COULTER, Tokyo, Japan) (Fig. 3A). The correlation coefficient is derived from the particles’ dynamic behavior in the sample, primarily Brownian motion. The scattered light intensity pattern changes as particles undergo Brownian motion over time. For particles before dispersion, the slow decay of the correlation coefficient indicates the formation of large clusters in the suspension. Conversely, dispersed particles show rapid decay of the correlation coefficient, indicating small, uniformly sized particles in the suspension. The majority of PAA particles measured by DLS were within the size range of 10–1000 nm, with a peak distribution occurring around 10–50 nm (Fig. 3B). Electron microscopy analysis revealed primary particle diameters of 42.9 nm before dispersion and 22.5 nm after dispersion, with aggregate sizes of 153.0 nm before dispersion and 28.5 nm after dispersion in the 2.5 mg/mL suspension. The primary particle diameters before and after dispersion, as well as the aggregate particle diameter after dispersion, were in good agreement with the particle size measurement results obtained by DLS, confirming the accurate measurement of dispersed primary particles within the experimental error range.

**Table 1:** Physiochemical characterization of the polymer used in the present study.

**Structural formula**	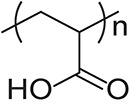
**Weight average molecular weight (MW)**	7.65 × 10^5^
**Number average molecular weight (Mn)**	4.14 × 10^5^
**Poly dispersity index (PDI)**	1.85
**Degree of crosslinking**	∼0.1%
**radius of gyration (Rg)**	68.7 nm

### 3.2 Relative lung weights

**Fig. 4:**
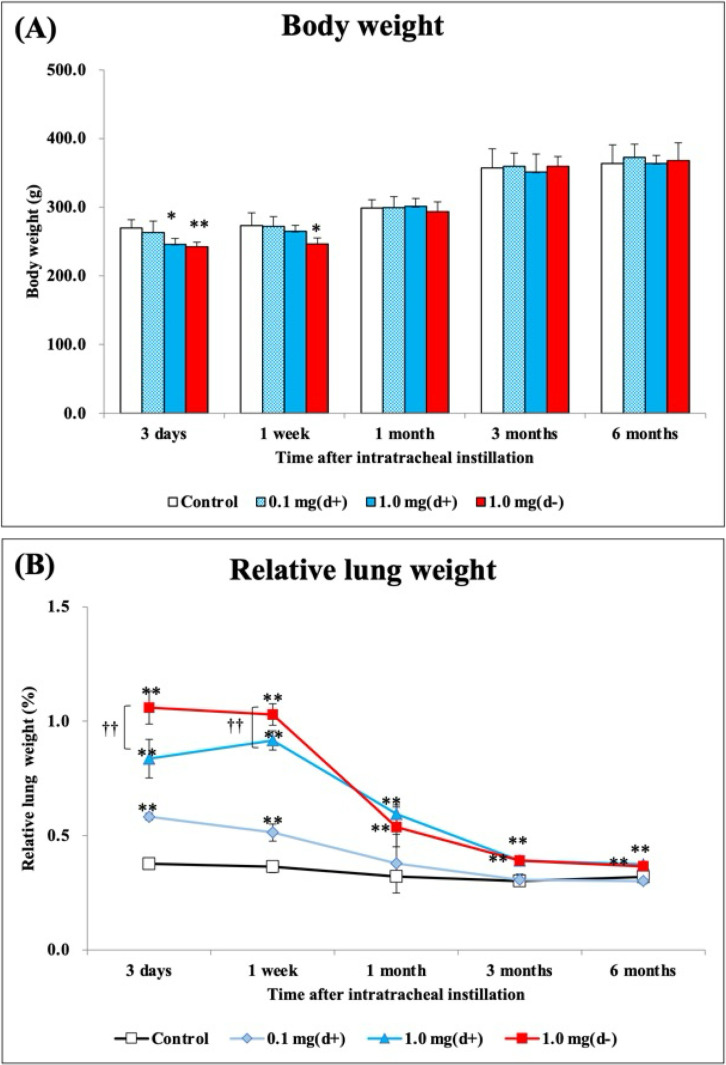
Body weight and relative lung weight after intratracheal instillation. (A) Time course of changes in the body weights of rats in each group. (B) Relative weight of the whole lung was calculated as a ratio of whole lung weight (g) to body weight (g) for each rat. The relative lung weight exhibited a dose-dependent increase during the observation period. All data are presented as mean ± SE (* p < 0.05, ** p < 0.01 indicate that the values are significantly higher than the control group; † p < 0.05, †† p < 0.01 indicate that the values are significantly higher than in the 1.0 mg dispersion group).

In both the 1.0 mg dispersion and 1.0 mg non-dispersion groups, the body weight decreased from 3 days to 1 week post-exposure compared to the control group. There were no significant differences in body weight among the dispersion and non-dispersion groups (Fig. 4A). The relative lung weight (lung weight/body weight) exhibited a dose-dependent increase during the observation period. There was a significant difference in relative lung weight between the 1.0 mg dispersion and 1.0 mg non-dispersion groups from 3 days to 1 week post-exposure (Fig. 4B).

### 3.3 Cell analysis and total protein and LDH activity in BALF

**Fig. 5:**
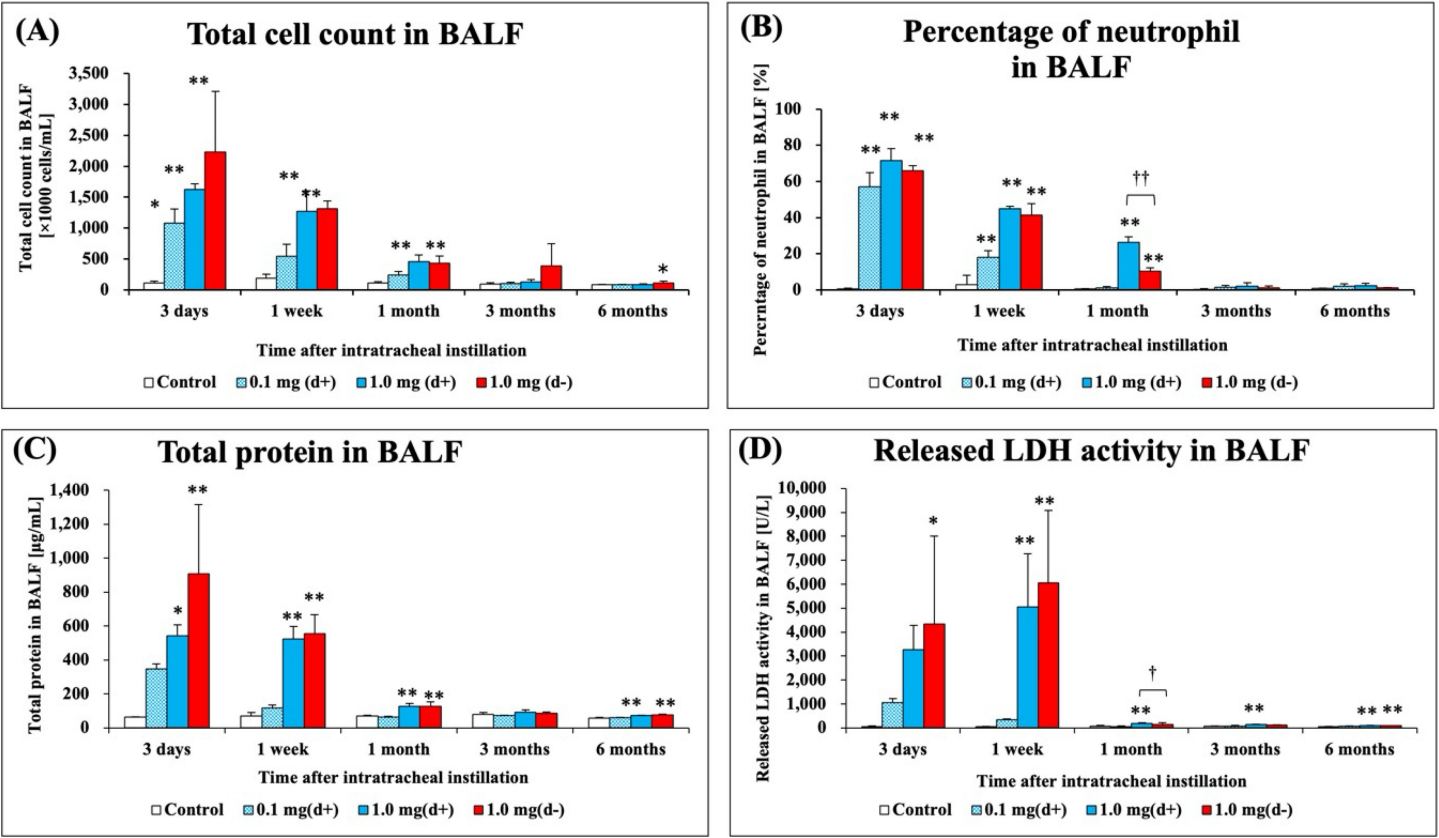
Analysis of cell number, total protein and released LDH activity in BALF following intratracheal instillation. (A) Total cell count in BALF, (B) Percentage of neutro-phil in BALF, (C) Total protein in BALF, (D) Released LDH activity in BALF. All data are presented as mean ± SE (* p < 0.05, ** p < 0.01 indicate that the values are significantly higher than the control group; † p < 0.05, †† p < 0.01 indicate that the values are significantly higher than in the 1.0 mg non-dispersion group).

Figure 5 shows the results of inflammatory cell counts, LDH activity, and total protein in BALF. There were statistically significant increases in the total cell count and percentage of neutrophils in both exposure groups from 3 days to 1 month compared with each control group (Fig. 5A–B). The results of total protein in BALF, an index of vascular permeability factor, showed statistically significant increases from 3 days to 1 month and 6 months in both exposure groups (Fig. 5C). The results of released LDH activity levels, an index of cytotoxicity, also showed statistically significant increases during the observation period in both exposed groups (Fig. 5D). There was a significant difference in total protein and released LDH activity levels in BALF between the 1.0 mg dispersion and 1.0 mg non-dispersion groups at 1 month post-exposure.

### 3.4 Micro-Computed Tomography (CT) imaging

**Fig. 6:**
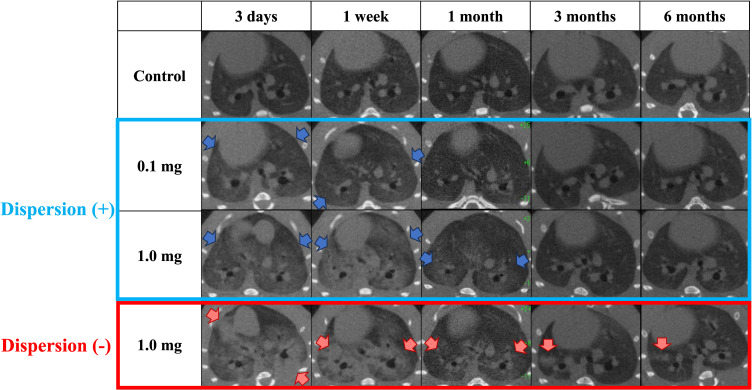
3D micro-CT imaging post-intratracheal instillation: visualization method and anatomical correlation. Diffuse or centrilobular infiltration in both lungs was present in a dose-dependent manner at 3 days and 1 month after exposure. In the dispersion group, ground-glass opacities were observed relatively uniformly and diffusely (blue arrows), while the non-dispersion group showed heterogeneous distribution of consolidation and ground-glass opacities (red arrows).

Centrilobular infiltration was revealed in the lungs from 3 days to 1 month after exposure in a dose-dependent manner. An improvement in lung infiltrations was observed at 3 months as compared with those at 3 days and 1 month after intratracheal instillation of PAA. In the non-dispersion group, consolidation and ground-glass opacity were observed with a heterogeneous distribution. In the dispersion group, ground-glass opacity was observed relatively uniformly and diffusely (Fig. 6).

### 3.5 Histopathological features in the lung

**Fig. 7:**
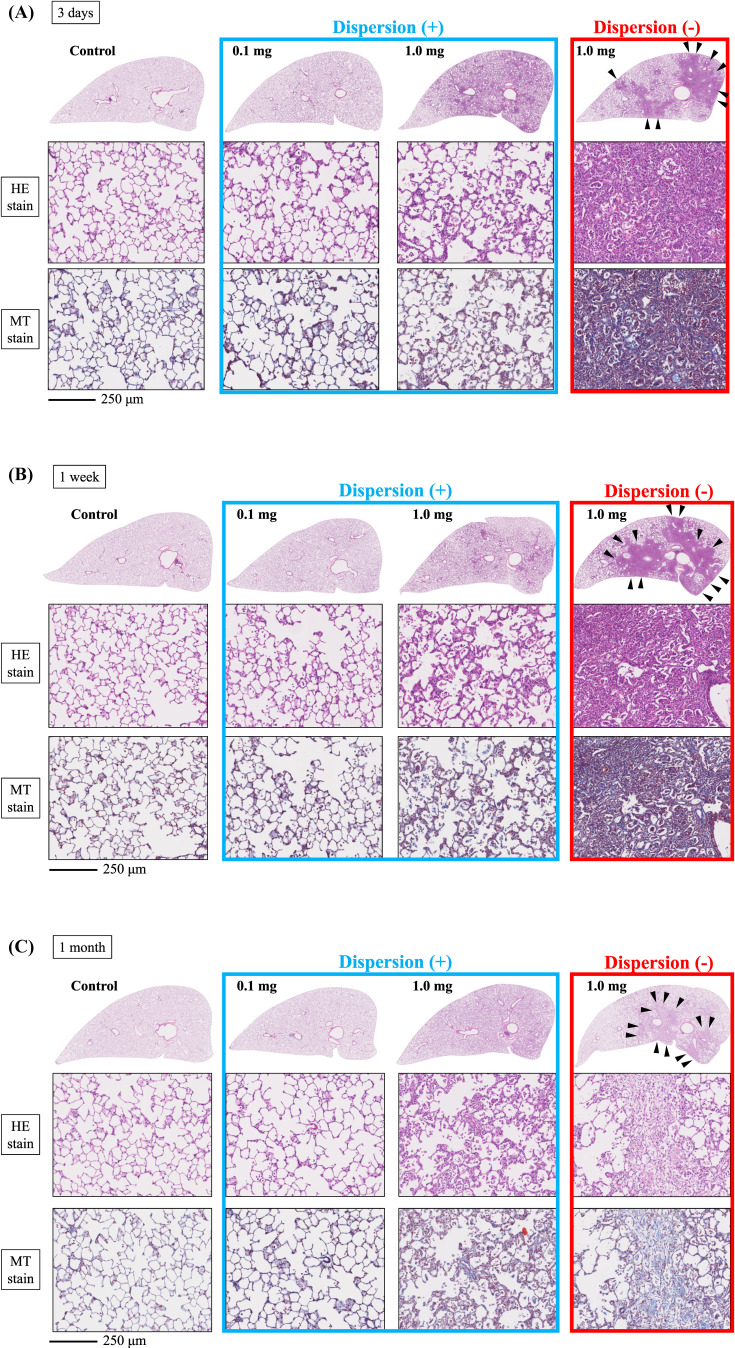

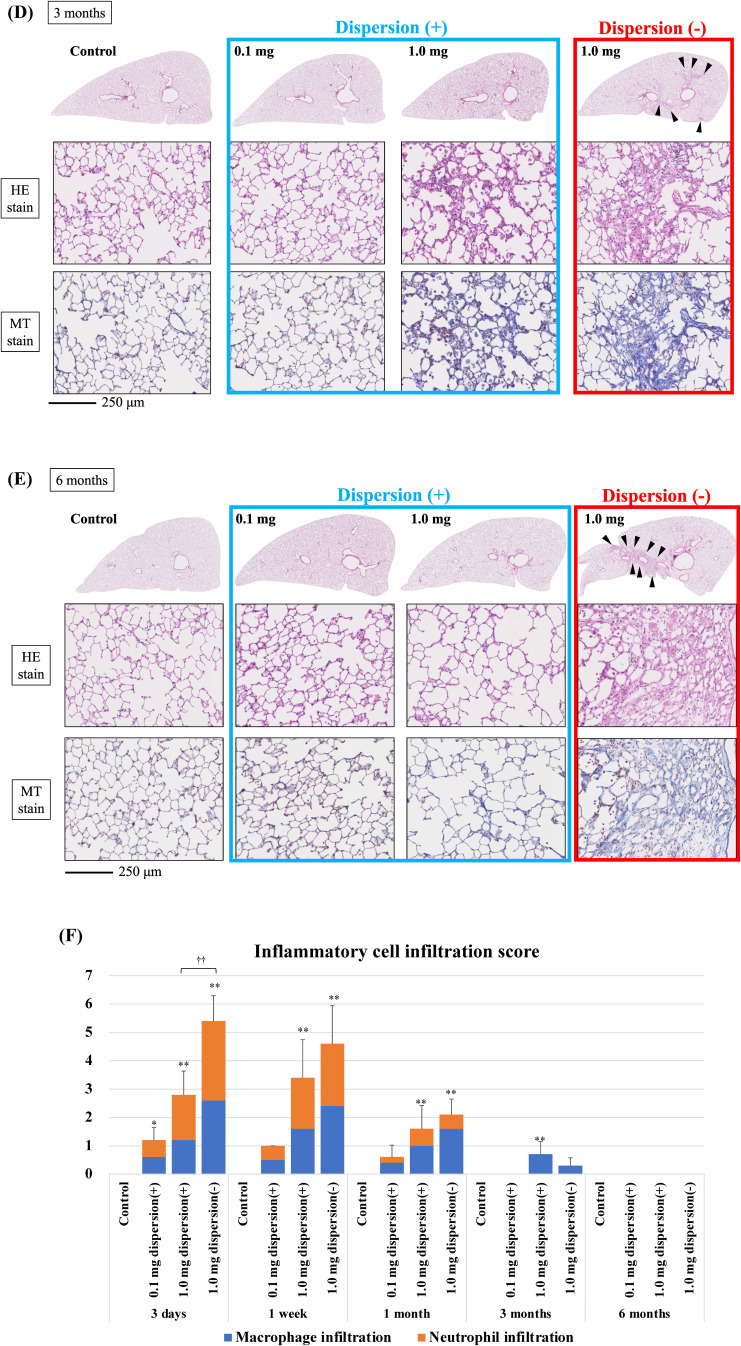

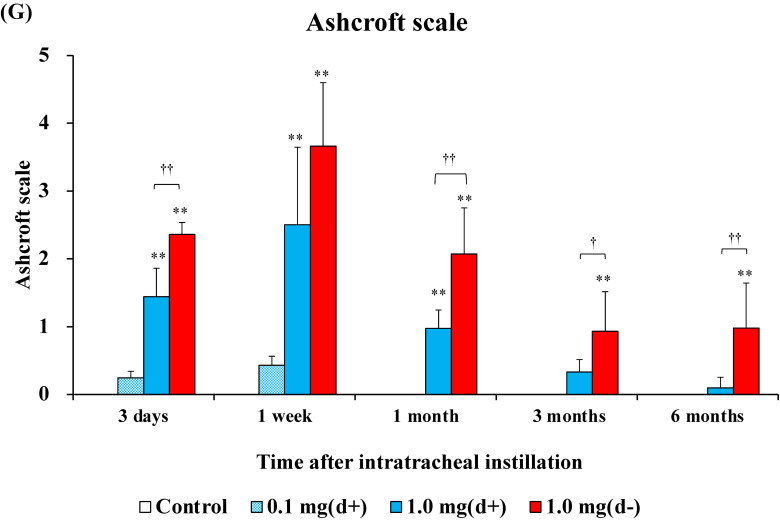
Histological findings after intratracheal instillation (hematoxylin and eosin (HE) and Masson’s trichrome (MT) staining). (A) Histological findings with HE staining and MT staining at 3 days, (B) 1 week, (C) 1 month, (D) 3 months and (E) 6 months after intratracheal instillation. The scale of the black bar is 250 µm. In the dispersion group, no inflammatory masses were observed, and the distribution was relatively uniform. In contrast, inflammatory masses (black arrow heads) were observed only in the non-dispersion group. (F) Inflammatory scale at each time point. (G) Ashcroft scale at each time point is presented as mean ± SE (* p < 0.05, ** p < 0.01 indicate that the values are significantly higher than the control group; † p < 0.05, †† p < 0.01 indicate that the values are significantly higher than in the 1.0 mg dispersion group).

Histopathological findings in the lung following intratracheal instillation of PAA are shown in Fig. 7. Inflammatory cell infiltration and edema were observed in the bronchial wall, predominantly neutrophils, all of which showed a dose-dependent pattern. These alterations reached their peak at 3 days and persisted up to 1 month, with a gradual tendency to diminish thereafter. Additionally, fibroblasts and collagen fibrils were observed in a dose-dependent manner, peaking at 1 week and tending to gradually decrease afterward (Fig. 7A–E). As indicated by the inflammatory scale, there was a notable dose-dependent elevation observed from day 3 onwards: it persisted for up to 1 month post-exposure in the non-dispersion group, while it peaked at 1 week and persisted to 3 months in the dispersion group (Fig. 7F). The fibrosis score, assessed by the Ashcroft scale, demonstrated a significant dose-dependent increase from 3 days, persisting up to 6 months post-exposure in the non-dispersion group, while it persisted up to 1 month in the dispersion group (Fig. 7G).

A centrilobular distribution was observed in both the non-dispersion and dispersion groups. In the non-dispersion group, there was almost no distribution in the apex and pleura, and inflammatory masses were seen predominantly in the lower lobes. No inflammatory masses were observed in the dispersion group, and the distribution was relatively uniform, with the spread of inflammation to the apex and pleura.

## 4. Discussion

The main findings obtained in the present study are as follows: (1) the molecular dispersion method suppressed aggregation of the chemical substance; (2) the intratracheal instillation using the molecular dispersion method exposed the lungs to PAA relatively uniformly and at the inhalation exposure level; (3) PAA caused inflammation in a dose-dependent manner and induced subsequent lung fibrosis.

In the present study, the molecular dispersion method was used to disperse PAA, which is prone to aggregation, generating a cluster-free suspension. As the result, all particles were less than 10 µm, with the majority measuring below 5 µm, indicating that these particles are within the respirable size range suitable for inhalation.

**Fig. 8:**
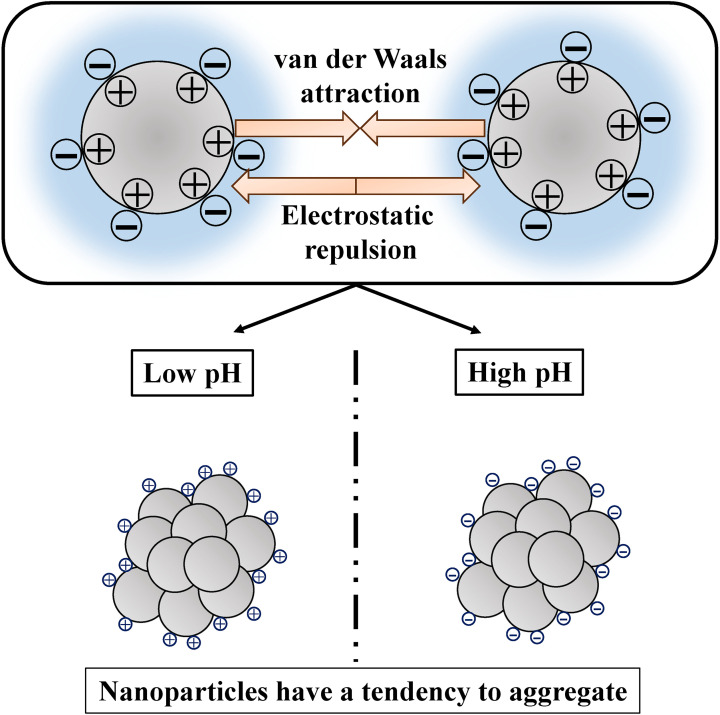
Nanoparticles naturally aggregate into clusters several microns in size. Their surfaces typically contain functional groups that react with H+ or OH−, causing them to acquire positive charges in acidic conditions and negative charges in basic conditions.

Nanoparticles tend to aggregate into clusters up to several microns in size [12–16]. Recent studies have applied principles of colloid science, based on the Derjaguin-Landau-Verwey-Overbeek (DLVO) theory, to understand nanoparticle aggregation under various conditions [17]. Most nanoparticle surfaces contain titratable surface functional groups that react with H^+^ or OH^−^. Particle surfaces typically acquire positive charges at low pH (excess H^+^), while the surfaces become negatively charged at high pH (excess OH^−^). The concentrations of H^+^ and OH^−^ reach equilibrium at a specific pH; this pH is termed the point of zero charge (PZC) or the zeta potential isoelectric point. As pH deviates from the PZC, the electric double-layer repulsive force increases while the relative van der Waals force decreases, promoting particle dispersion [18]. This property suggests that chemical aggregation can be controlled through pH modification (Fig. 8).

Although direct particle distribution was not observed in the present study, the distribution pattern of inflammatory cells served as a proxy indicator. Using the molecular dispersion method, PAA demonstrated relatively uniform centrilobular distribution in rat lungs, as evidenced by CT and pathological images. The pathological findings showed minimal inflammatory accumulation, with inflammation dispersed uniformly throughout the peripheral regions, including the apex and pleural areas. In contrast, conventional intratracheal instillation without dispersion resulted in centrilobular PAA deposition, predominantly in the central bronchi and lower lobes, with minimal lesions in the apex or pleural regions, indicating non-uniform distribution throughout the lung.

Previous studies have reported that intratracheal instillation leads to lesion concentrations primarily near the hilum and in peribronchiolar alveoli, with minimal subpleural involvement, resulting in heterogeneous distribution [19]. In inhalation exposure, which better simulates human exposure patterns, inflammation maintains a centrilobular pattern but exhibits relatively uniform distribution to the peripheral regions, including the apical and pleural areas (Supplemental). The similarity between the inflammatory cell distribution patterns observed in this study and those in inhalation exposure studies suggests comparable particle deposition patterns between both methods.

According to studies using CT-based human airway models, 2.5 µm particles showed the most uniform distribution, reaching the peripheral regions of the lungs [20]. Based on human inhalation exposure patterns, coal workers’ pneumoconiosis shows lesions distributed throughout the entire lung rather than being limited to the peripheral regions, presenting a centrilobular pattern [21]. The dispersion method used in this study therefore not only serves for inhalation exposure experiments but also may reflect the pathophysiology of inhaled chemical substances in the lungs, as it demonstrates similarities to human pulmonary deposition patterns.

In the present study, particles prepared using the molecular dispersion method exhibited a slightly lower quantitative inflammatory and fibrotic response in the lungs compared to aggregated particles, though the effect remained persistent. Previous studies have documented how differences in the aggregation and dispersion states of intratracheally instilled substances, including nanoparticles prepared by various dispersion methods, influence pulmonary reactivity. While dispersed nanoparticles induce collagen deposition in the alveolar walls, aggregated nanoparticles promote granuloma formation, resulting in qualitative differences in inflammatory responses [22] and also show persistent inflammation for more than a month, leading to pulmonary fibrosis [6]. On the other hand, in the intratracheal instillation of TiO_2_ and ZnO, which have low lung inflammation and fibrotic potential, the inflammation caused by intratracheal instillation was transient [23]. Other reports have also shown that intratracheal instillation of polyhexamethyleneguanidine phosphate (PHMG-P) (1.2 mg/kg BW, single exposure) in mice results in severe and persistent inflammation until 1 month [24]. The findings of those studies are similar to our previous research, indicating that chemical substances possessing pro-inflammatory and fibrogenic abilities elicit sustained inflammation, leading to the development of fibrosis with a similar pathological phenotype. In comparison with substances with known inflammogenicity and fibrogenicity, PAA induced pulmonary inflammation and fibrosis, suggesting its potential to cause lung damage. In non-dispersed PAA, inflammatory clusters formed in the lungs, resulting in more pronounced fibrosis in specific areas. This suggests that larger clusters exhibit increased water absorption capacity, leading to airway obstruction and subsequent formation of inflammatory clusters in our previous study [25].

The dispersion method used in this study employs charge-based particle surface modification through pH adjustment [26, 27]. Among other nanoparticle dispersion methods, mechanical approaches such as sonication are commonly used. However, mechanical dispersion methods like sonication can physically alter the physicochemical properties of the dispersed materials, which has been identified as a potential source of variability in results [28]. While inorganic particles may be less affected, polymeric compounds (including PAA) with molecular structures can potentially undergo molecular weight reduction and changes in intermolecular bonds during mechanical processing [29]. Therefore, mechanical dispersion methods may result in irregular molecular fragmentation, leading to variations in molecular weight, which raises concerns about uniform molecular weight exposure to the lungs. The dispersion method employed in this study does not involve physical processing such as mechanical grinding, suggesting that the physicochemical properties of the materials are maintained during dispersion. This method is particularly advantageous for dispersing organic substances, especially polymeric compounds. Intrapulmonary dispersion of PAA particles following intratracheal instillation studies using the molecular dispersion method was similar to that following inhalation studies.

In the present study, different solvent preparations were used across experimental groups to accommodate the specific requirements of PAA administration. For the molecular dispersion method, PAA was initially dissolved in NaOH and subsequently neutralized with HCl. This neutralization reaction (NaOH + HCl → NaCl + H_2_O) generates sodium chloride, which is identical to the salt component of normal saline used in both the control and non-dispersion groups. Consequently, equivalent osmolarity and ionic strength are maintained across all experimental solutions. This approach minimizes solvent-related confounding factors, ensuring that the observed biological effects can be attributed specifically to PAA exposure rather than differences in solution composition.

Particularly in terms of achieving relatively uniform substance distribution in the lungs when examining pulmonary inflammation and fibrosis. Future comparative studies between molecular dispersion-based intratracheal instillation and conventional intratracheal instillation using other chemical substances are expected to provide valuable insights into the roles of pulmonary inflammation, fibrosis, and particle deposition patterns.

## Supplementary information

**Additional file 1:** Histological findings after inhalation exposure at 3 days (hematoxylin and eosin (HE) staining).

## Declarations

### Notes

All procedures and animal handling were done according to the guidelines described in the Japanese Guide for the Care and Use of Laboratory Animals as approved by the Animal Care and Use Committee, University of Occupational and Environmental Health, Japan (animal studies ethics clearance proposal number: AE18-021). Consent for publication is not required as no human data is presented. The datasets during and/or analyzed during the current study are available from the corresponding author on reasonable request. The authors declare no competing financial interest.

### Availability of data and material

Data available on request from authors.
