# Supplementary material for: Development of a new alternative method to inhalation exposure: intratracheal instillation studies using molecular dispersion
Source: Environ Health Prev Med. 2025 Sep 11;30:69. doi: 10.1265/ehpm.25-00142 (PMC12436071; doi:10.1265/ehpm.25-00142)
Supplement: Supplementary file 1 — Additional file 1: Histological findings after inhalation exposure at 3 days (hematoxylin and eosin (HE) staining). [file ehpm-30-069-s001.docx]

Histological findings after inhalation exposure at 3 days (hematoxylin and eosin (HE) staining). Rats (F344) were exposed to PAA at a concentration of 2.0 mg/m³ for 6 hours per day, 5 days per week.

The inhalation exposure method, which better reflects human exposure scenarios, resulted in inflammation that was both centrilobular and uniformly distributed throughout the peripheral regions, including the apical and pleural areas. For comparison, histological findings following intratracheal instillation of PAA using molecular dispersion are also shown, demonstrating the relatively similar uniform distribution pattern observed with the inhalation exposure.
